# Supplementary material for: Photocatalytic Isomerization of (E)-Anethole to (Z)-Anethole
Source: Molecules. 2022 Aug 22;27(16):5342. doi: 10.3390/molecules27165342 (PMC9412280; doi:10.3390/molecules27165342)
Supplement: Supplementary file 1 [file molecules-27-05342-s001.zip › molecules-1874169-supplementary.pdf]

Supplementary Materials

Photocatalytic Isomerization of (*E*)-Anethole to (*Z*)-Anethole

Marvin Korff [1,2], Tiffany O. Paulisch [1,3], Frank Glorius [1,3], Nikos L. Doltsinis [4] and Bernhard Wünsch [1,2,*]

[1] Westfälische Wilhelms-Universität Münster, Chemical biology of ion channels (Chembion) , GRK 2515, Corrensstraße 48, D-48149 Münster, Germany

[2] Westfälische Wilhelms-Universität Münster, Institut für Pharmazeutische und Medizinische Chemie, Corrensstraße 48, D-48149 Münster, Germany

[3] Westfälische Wilhelms-Universität Münster, Organisch-Chemisches Institut, Corrensstraße 40, D-48149 Münster, Germany

[4] Westfälische Wilhelms-Universität Münster, Institut für Festkörpertheorie und Center for Multiscale Modelling and Computation, Wilhelm-Klemm-Straße 10, D-48149 Münster, Germany

***** Correspondence: wuensch@uni-muenster.de; Tel.: +49-251-8333311; Fax: +49-251-8332144

Table of Contents

1. General Methods S3

2. Setup of the Photoreactor S4

3. Calculations of triplet energies S5

4. Determination of the conversion of (*E*)-anethole by ^{1}H NMR spectroscopy S5

5. Further Experiments S7

6. Structures of the photosensitizers used in this study S9

7. Recycling experiment S10

8. Photostationary state experiment S10

9. Suzuki-Miyaura coupling to prepare (*Z*)-anethole S11

10. NMR spectra S13

11. References S16

1. General Methods

Solvents and reagents: Unless otherwise mentioned, solvents and commercial reagents and compounds were used without further purification. THF was dried with sodium/benzophenone and was freshly distilled before use.

Thin-layer chromatography (TLC): silica gel 60 F_{254} plates (Merck).

Flash chromatography (FC): silica gel 60, 40–63 μm (Macherey-Nagel).

Automated flash column chromatography: Isolera One (Biotage).

Melting point: Determination *via* melting point apparatus MP50 (Mettler Toledo) using an open capillary.

NMR spectroscopy: ^{1}H NMR (400 MHz, 600 MHz), and ^{13}C NMR (100 MHz, 151 MHz): Agilent DD2 400 and 600 MHz spectrometers; the temperature of all samples was set to 299 K; Chemical shifts (δ) are reported in parts per million (ppm) and are referenced to tetramethylsilane (TMS) and the used deuterated solvent. Analyzation of the spectra was performed using the MestReNova software.

IR spectroscopy: Spectra were recorded on a MIRacle 10 device (Shimadzu), using the IRsolution software. All samples were applied to the device without solvent and were directly measured. The absorption bands are characterized by their wavenumbers ($\tilde{\nu}$).

High resolution mass spectrometry (HRMS): All samples were measured in the positive ion mode. Therefore, all specified molecules represent positively charged ions. Atmospheric pressure chemical ionization (APCI) mass spectra were recorded with a MicroTOFQ mass spectrometer (Bruker). Data were analyzed with the DataAnalysis software. Deviations of the found exact masses from the calculated exact masses were 5 mDa or less. The DirectProbe/APCI-source was operated in the positive ionization mode, scan range *m*/*z* = 60–1000. APCI-conditions: The capillary was set to 4.0 kV, the nebulizer was operated at 0.7 bar, the dry gas was set to 3.0 L/min at a temperature of 200 °C. Mass calibration was done using a Fatty Acid Methyl Esters (FAME) solution (*m*/*z* = 103–383 or 423–565) in dichloromethane.

2. Setup of the Photoreactor

For the purpose of this study, a photoreactor was built in our in-house workshop. (cf. Figure S1) The interior of a metal can was coated with 5 m of LED strip, comprising 600 LEDs in total. Each LED has a power of 80 mW, leading to a total power of 48 W in the photoreactor. The excitation maximum of the LEDs is at 400 nm. For temperature control, holes were put in the bottom and lid of the metal can. On the bottom, a channel connects the can with a ventilator, which was installed on the side of the device instead directly beneath, to prevent interference of the fan with the magnetic stirrer. Under performance, the interior temperature of the photoreactor reaches a consistent temperature of 28 °C. All in all, this photoreactor was cheap to assemble, with a cost of about 100 $ in materials.

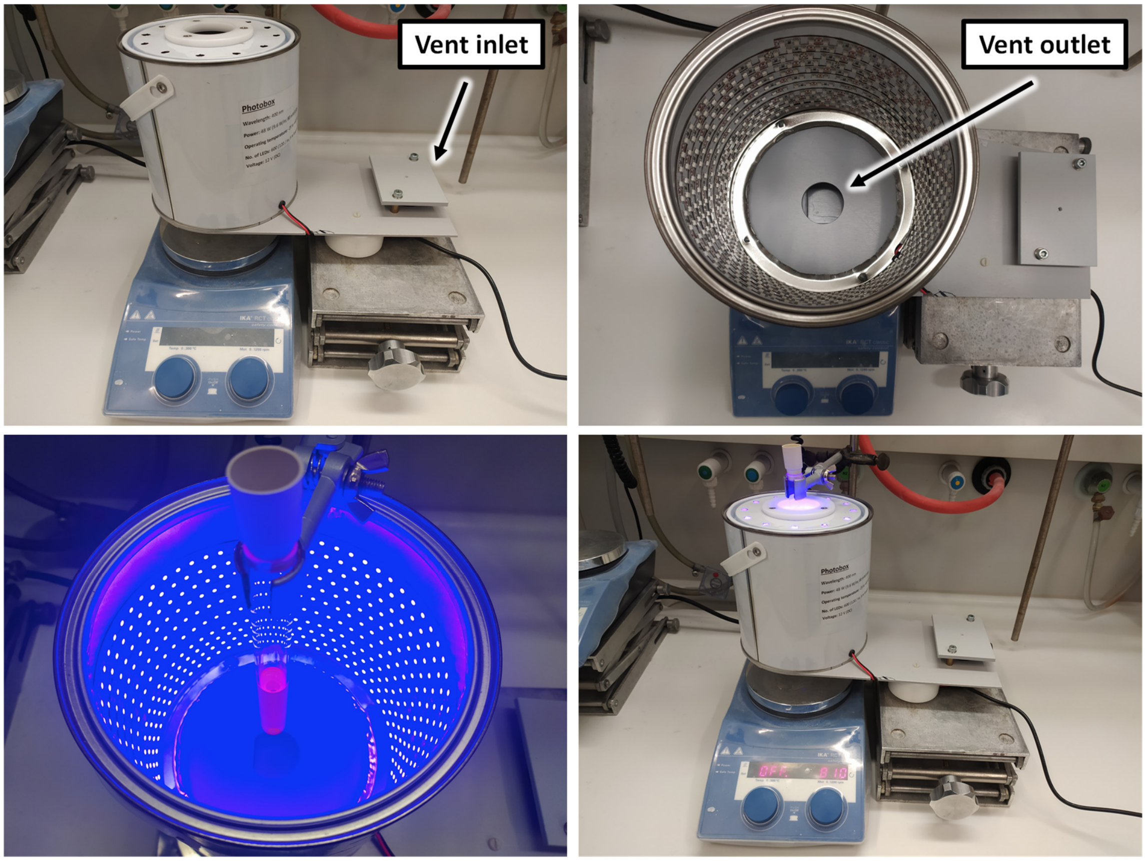

Figure S1: Setup of the photoreactor. Top left: Photoreactor placed on a magnetic stirrer, the cooling fan is attached to the side of the apparatus. Top right: the interior wall of the photoreactor is covered with LED strips and an air canal from the fan is connected to a hole in the bottom of the reactor. Bottom left: Operating photoreactor with opened lid and a reaction mixture placed inside. Bottom right: Typical operation of the photoreactor with closed lid.

3. Calculations of triplet energies

The geometries of (*E*)-anethole and (*Z*)-anethole were optimized in their respective local minima in the T1 state using DFT with the B3LYP hybrid functional [1], with a 6-31G* basis set [2–4]. The T_1 state was calculated within the standard Kohn-Sham approach with spin multiplicity 3, 0-0 excitation energies for the $S_0 \rightarrow T_1$ transition by subtracting the energy of the respective S_0 optimized geometry from the corresponding T_1 local minimum energies. All calculations were performed using the Gaussian 09 Rev. D.01 quantum chemistry package [5]. Solvent effects were taken into account using the polarizable continuum model (PCM) [6], with UFF atomic radii for acetonitrile [7].

4. Determination of the conversion of (*E*)-anethole by ^{1}H NMR spectroscopy

All ^{1}H NMR spectra were phase- and baseline-corrected prior to analysis to ensure exact and comparable integration. To determine the conversion of the photocatalyzed isomerization of (*E*)-anethole to (*Z*)-anethole by ^{1}H NMR spectroscopy, a pair of isolated signals from both compounds had to be identified in the spectrum. Therefore, the ^{1}H NMR spectra of pure (*E*)-anethole and the non-purified reaction product were overlayed. (Figure S2) The proton in 2-position of the propenyl substituent proved to be well-suited for this analysis. The signal of 2-H of (*E*)-anethole (δ = 6.11 ppm) and (*Z*)-anethole (δ = 5.66 ppm) were integrated, and the sum was set to 100%. The conversion (in %) was defined as integral of 2-H ((*Z*)-**1**) divided by the sum of integrals multiplied by 100%. Furthermore, the spectra of the crude products were carefully analyzed for additional signals resulting from potentially side products. This analysis allows the evaluation of the transformation and the purity of the products.

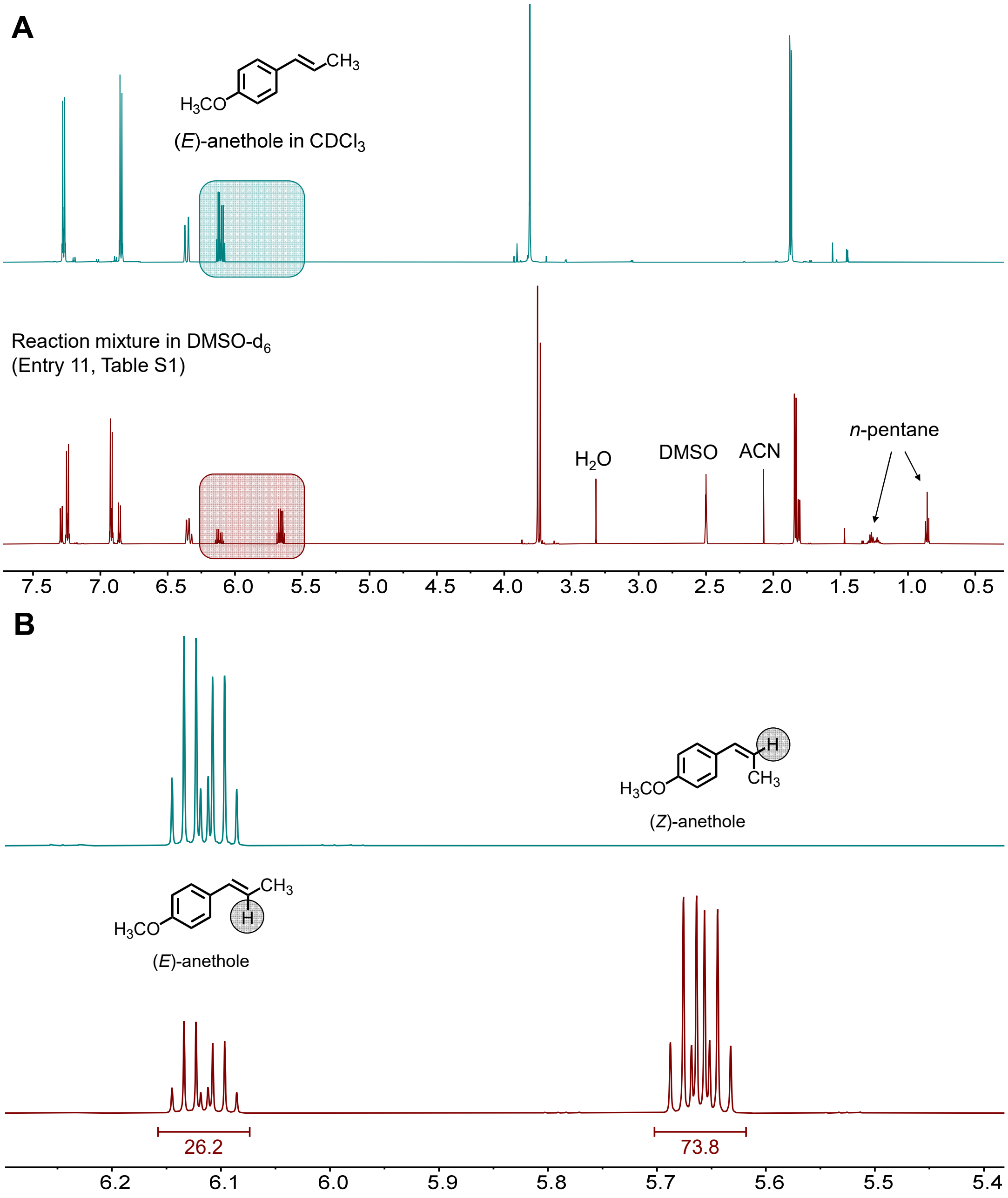

Figure S2: ^{1}H NMR spectroscopy to determine the reaction conversion of (*E*)- to (*Z*)-anethole **A**: Overlayed ^{1}H NMR spectra of (*E*)-anethole (in $CDCl_3$, top) and mixture of (*E*)-anethole and (*Z*)-anethole (in DMSO-d_6, bottom). The signals stemming from 2-H are marked in a green and red box, respectively. **B**: Expansion of the 2-H signal in the ^{1}H NMR spectrum of (*E*)-anethole (in $CDCl_3$, top) showing a quartet of a doublet (dq). Expansion of the 2-H signals in the ^{1}H NMR spectrum of a mixture of both isomers (in DMSO-d_6, bottom). Both signals are quartets of doublets (dq). The determined reaction conversion in this case is 74%.

5. Further Experiments

Table S1: Screening of photosensitizers regarding their potential to convert (*E*)- into (*Z*)-anethole. Organo-photosensitizers were used in an amount of 20 mol% and metal-based photosensitizers in an amount of 2 mol%, relative to (*E*)-anethole. The transformations were determined by recording ^1H NMR spectra of the crude products.

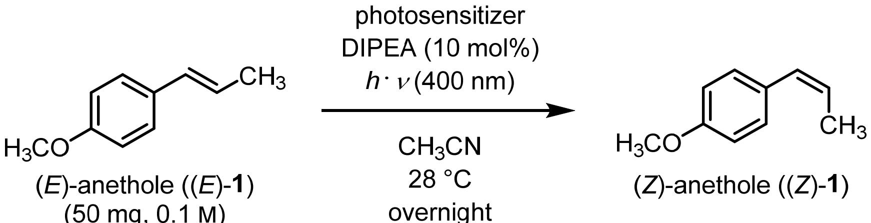

(*E*)-anethole ((*E*)-**1**) (50 mg, 0.1 M) → (*Z*)-anethole ((*Z*)-**1**)

entry	photosensitizer	E_T (PS) [kcal/mol]	ratio (***E***) : (***Z***)
1	-	-	100 : 0
2	$[Ru(dmbpy)_3](PF_6)_2$	45.3	100 : 0
3	$[Ru(bpy)_3](BF_4)_2$	46.5	100 : 0
4	[Ir(d*t*bbpy)(ppy)$_2$](PF$_6$)	49.2	15 : 85
5	Riboflavin	49.9	100 : 0
5[a]	Riboflavin	49.9	100 : 0
6	Benzil	54.2	99 : 1
6[a, b]	Benzil	54.2	87 : 13
6[a, b, c]	Benzil	54.2	94 : 6
7[a, b, d]	Benzil	54.2	-
7	Ir(*p*-*t*Bu-ppy)$_3$	54.5	10 : 90
8	$Ir(ppy)_3$	55.2	12 : 88
9	Ir(*p*-CF$_3$-ppy)$_3$	56.4	11 : 89
10[a]	Chrysene	57.1	93 : 7
11	[Ir(dFCF$_3$ppy)$_2$(d*t*bpy)]PF$_6$	60.1	26 : 74
12	Thioxanthone	63.3	50 : 50

[a] without DIPEA
[b] considerable amounts of side products were observed
[c] benzene instead of CH_3CN
[d] 1.0 eq. of benzil

The given triplet energy values of the catalysts are not absolute values, as they were measured under different conditions. For chemical structures of photosensitizers and references of triplet energies see Table S3.

Table S2: Optimization of reaction conditions for the photoisomerization of (*E*)-anethole into (*Z*)-anethole using Ir(*p*-*t*Bu-ppy)$_3$ as photosensitizer. The conversion was determined by ^{1}H NMR spectroscopy of non-purified products.

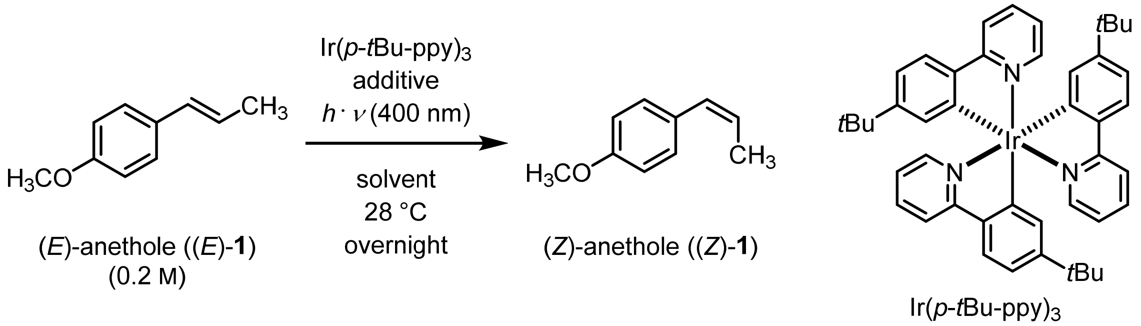

entry	conc. [mol%] Ir(*p*-*t*Bu-ppy)$_3$	additive	solvent	ratio (*E*) : (*Z*)	substrate scale
1[a]	2	DIPEA (0.1 eq.)	CH_3CN	10 : 90	50 mg
2[a]	1	DIPEA (0.1 eq.)	CH_3CN	10 : 90	100 mg
3	1	DIPEA (0.1 eq.)	CH_3CN	10 : 90	100 mg
4	0.5	DIPEA (0.1 eq.)	CH_3CN	10 : 90	200 mg
5	0.1	DIPEA (0.1 eq.)	CH_3CN	10 : 90	1.00 g
6	0.1	-	CH_3CN	10 : 90	1.00 g
7	0.1	-	DCM	10 : 90	1.00 g
8	0.1	-	THF	10 : 90	1.00 g
9	0.1	-	MeOH	10 : 90	1.00 g
10[b]	0.1	-	MeOH	10 : 90	630 mg
11[c]	0.1	-	MeOH	25 : 75	1.00 g

[a] concentration of *E*-anethole was 0.1 M.

[b] photosensitizer of entry 9 was recovered *via* flash chromatography (63%) and reused.

[c] reaction time decreased to 8 h.

6. Structures of the photosensitizers used in this study

[Ru(dmbpy)₃](PF₆)₂

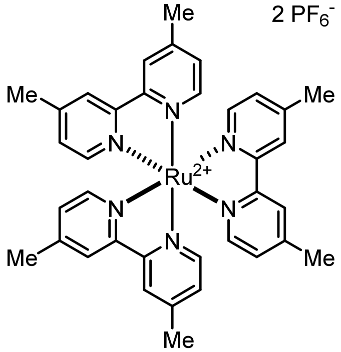

E_T = 45.3 kcal/mol [8]

[Ru(bpy)₃](BF₄)₂

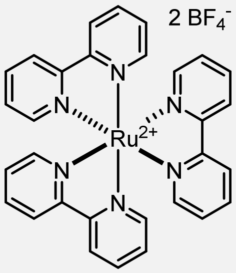

E_T = 46.5 kcal/mol [8]

[Ir(dtbbpy)(ppy)₂]PF₆

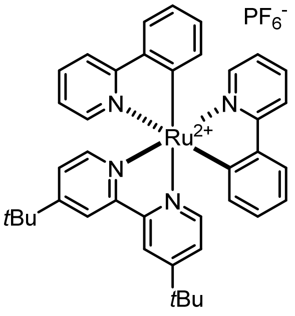

E_T = 49.2 kcal/mol [8]

Riboflavin

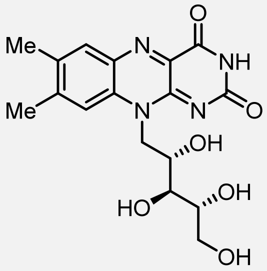

E_T = 49.9 kcal/mol [9]

Benzil

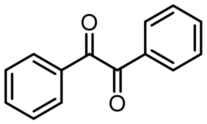

E_T = 54.2 kcal/mol [9]

Ir(*p*-*t*Bu-ppy)₃

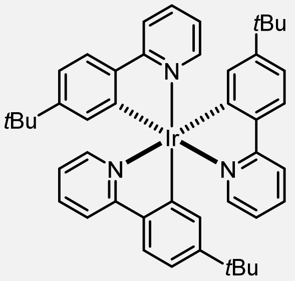

E_T = 54.5 kcal/mol [10]

Ir(ppy)₃

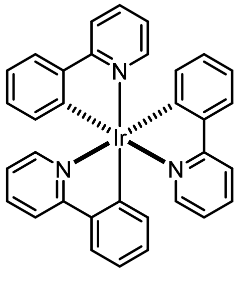

E_T = 55.2 kcal/mol [10]

Ir(*p*-CF₃-ppy)₃

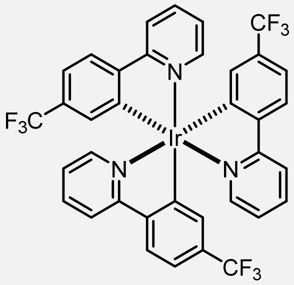

E_T = 56.4 kcal/mol [10]

Chrysene

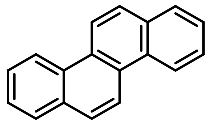

E_T = 57.1 kcal/mol [9]

[Ir(dFCF₃ppy)₂(dtbpy)]PF₆

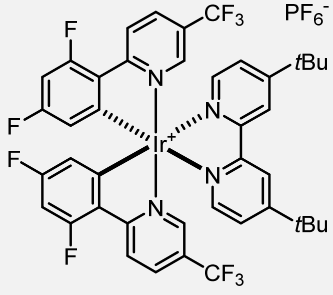

E_T = 60.1 kcal/mol [10]

Thioxanthone

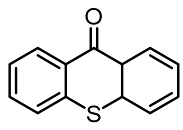

E_T = 63.3 kcal/mol [9]

7. Recycling experiment

In order to perform the recycling experiment (Table S2, entry 10), the photosensitizer Ir(*p*-*t*Bu-ppy)$_3$ used in entry 9, Table S2, was isolated from the crude reaction product *via* automated flash column chromatography (cyclohexane/EtOAc = 99:1 → 80:20). It was recovered in 63% yield (3.5 mg, 4.3 μmol) and reused in a subsequent photoreaction, scaled appropriately to the amount of photosensitizer. The reaction was performed analogously to the described general method.

8. Photostationary state experiment

To determine the photostationary state of the reaction, an additional photoreaction was performed under optimized conditions, analogously to the general procedure (Table S2, entry 11). In regular intervals, aliquots (equivalent to 10 mg substrate) were taken with a syringe from the reaction mixture under nitrogen atmosphere. The solvent of the aliquots was removed *in vacuo* and the residue was dissolved in deuterated DMSO and submitted for ^{1}H NMR analysis. The results are depicted in Figure S3.

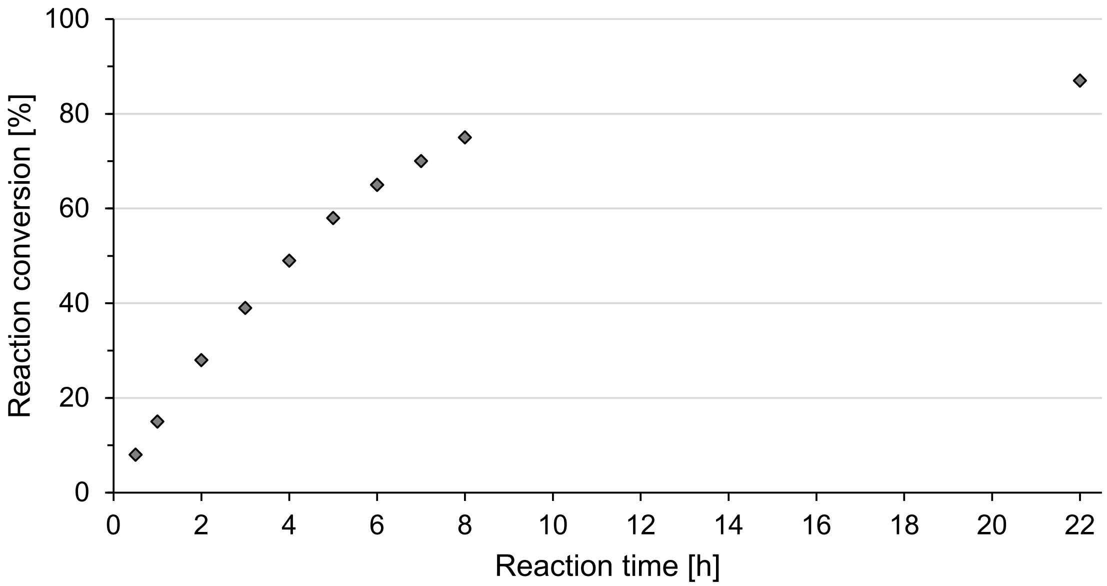

Reaction time [h]	0.5	1	2	3	4	5	6	7	8	22
Reaction conversion [%]	8	15	28	39	49	58	65	70	75	87

Figure S3: Determination of the reaction time required to reach the photostationary state. Aliquots of the reaction mixture were taken in regular intervals and the reaction conversion was determined by ^{1}H NMR spectroscopy.

9. Suzuki-Miyaura coupling to prepare (*Z*)-anethole

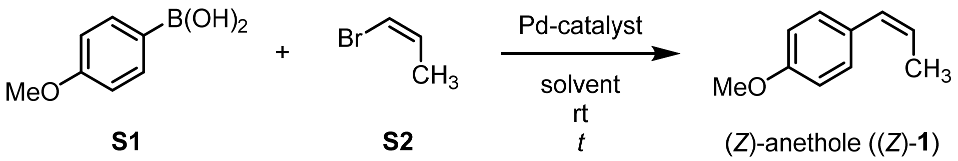

Scheme S1: Suzuki-Miyaura coupling of (4-methoxyphenyl)boronic acid (**S1**) and (*Z*)-1-bromoprop-1-ene (**S2**) to (*Z*)-anethole ((*Z*)-**1**).

$Pd(PPh_3)_3$ as catalyst

Under nitrogen atmosphere, (*Z*)-1-bromoprop-1-ene (**S2**, 100 mg, 826 µmol), (4-methoxyphenyl)boronic acid (**S1**, 188 mg, 1.24 mmol, 1.50 eq.), Cs_2CO_3 (539 mg, 1.65 mmol, 2.00 eq.) and $Pd(PPh_3)_3$ (76.4 mg, 66.1 µmol, 8 mol%) were suspended in THF (dry, 3 mL). The mixture was stirred at room temperature overnight. The mixture is diluted with EtOAc (10 mL) and washed with water (3 × 10 mL). The organic layer

was dried over Na_2SO_4 and the solvent was removed *in vacuo*. The crude product was purified *via* automated flash column chromatography (cyclohexane / EtOAc = 100 : 0 → 95 : 5). The product was obtained as a colorless liquid (3.9 mg, 26 µmol, 3% yield) as a mixture of (*Z*)-/(*E*)-anethole in a ratio of 73 : 27 (determined by ^{1}H NMR spectroscopy).

XPhos/XPhos G2 as catalyst system

Under nitrogen atmosphere, (*Z*)-1-bromoprop-1-ene (**S2**, 100 mg, 826 µmol), (4-methoxyphenyl)boronic acid (**S1**, 188 mg, 1.24 mmol, 1.50 eq.), XPhos G2 (13.0 mg, 16.5 µmol, 2 mol%), XPhos (7.9 mg, 17 µmol, 2 mol%), Cs_2CO_3 (539 mg, 1.65 mmol, 2.00 eq.) were dissolved in a mixture of THF (4 mL) and water (1 mL). The mixture was stirred at room temperature overnight. The mixture was diluted with diethyl ether (10 mL) and washed with water (3 × 10 mL). The organic layer was dried over Na_2SO_4 and the solvent was removed *in vacuo*. The crude product was purified *via* automated flash column chromatography (cyclohexane/EtOAc = 99 : 1 → 98 : 2). The product was obtained as a yellowish oil (43.7 mg, 295 µmol, 36% yield) as a mixture of (*Z*)-/(*E*)-anethole in a ratio of 94 : 6 (determined by ^{1}H NMR spectroscopy).

$Pd_2(dba)_3$/(*t*Bu)$_3$PHBF$_4$ as catalyst system

The synthesis was performed according to the descriptions of Netherton and Fu [11]. Under nitrogen atmosphere, (*Z*)-1-bromoprop-1-ene (**S2**, 200 mg, 1.65 mmol), (4-methoxyphenyl)boronic acid (**S1**, 276 mg, 1.82 mmol, 1.10 eq.), $Pd_2(dba)_3$ (16.7 mg, 16.5 µmol, 1 mol%), (*t*Bu)$_3$PHBF$_4$ (9.6 mg, 33 µmol, 2 mol%), KF (317 mg, 5.46 mmol, 3.3 eq.) were dissolved in THF (dry, 3 mL). The solution was stirred at room temperature for 3 h. The mixture was diluted with diethyl ether (25 mL) and filtrated over a pad of silica. The solvent was removed *in vacuo* and the crude product was purified via automated flash column chromatography (cyclohexane / EtOAc = 100 : 0 → 98 : 2). The product was obtained as a colorless liquid (184 mg, 1.24 mmol, 75% yield) as a mixture of (*Z*)-/(*E*)-anethole in a ratio of 62 : 38 (determined by ^{1}H NMR spectroscopy).

10. NMR spectra

^{1}H NMR spectrum

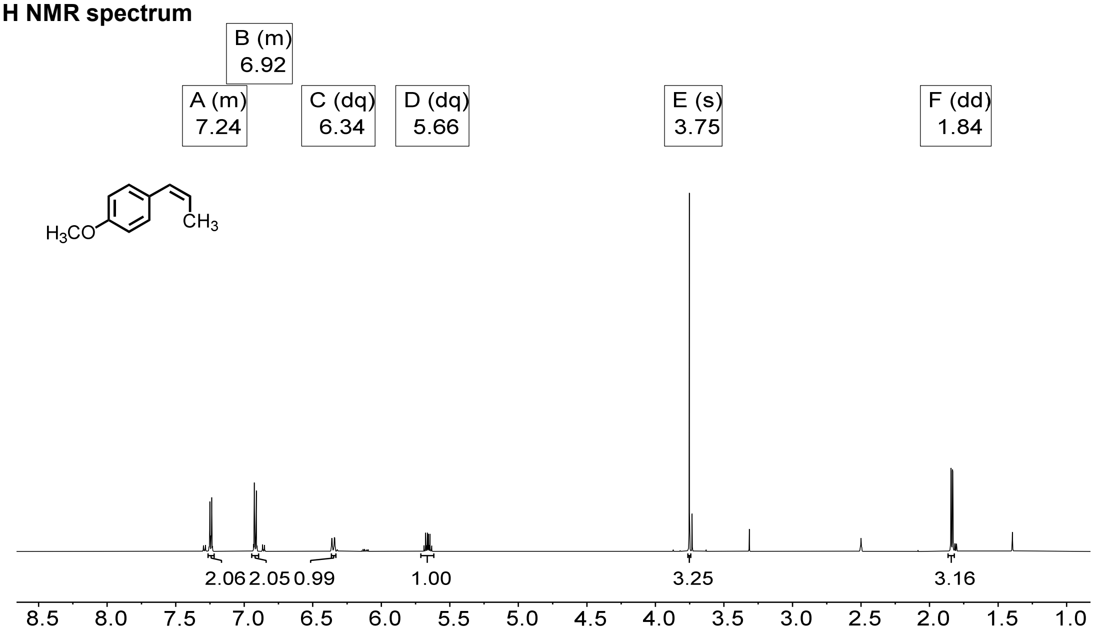

^{13}C NMR spectrum

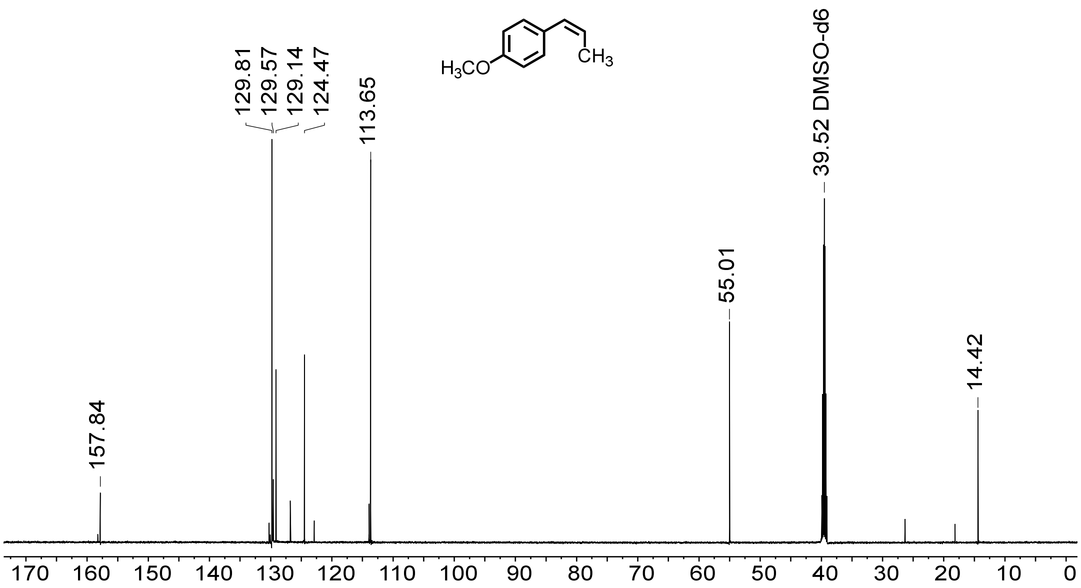

Figure S4: NMR spectra of (*Z*)-1-methoxy-4-(prop-1-en-1-yl)benzene ((*Z*)-anethole (*Z*)-**1**)).

^{1}H NMR spectrum

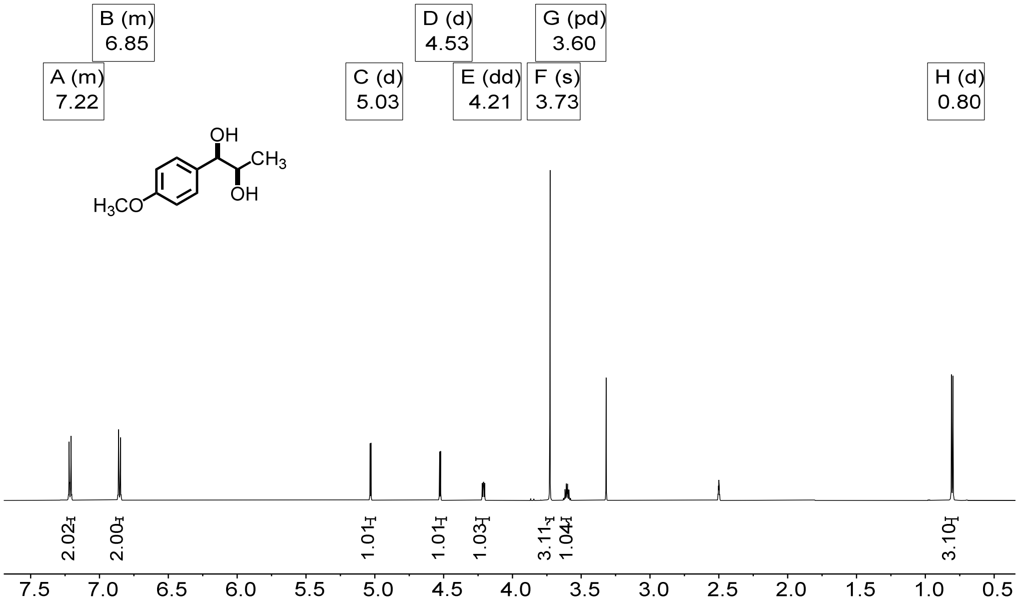

^{13}C NMR spectrum

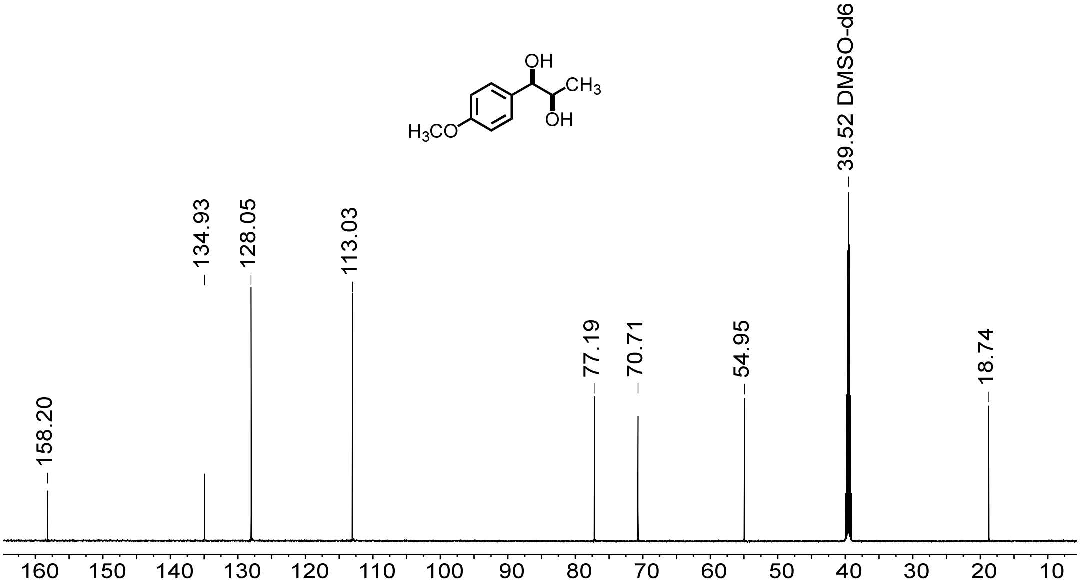

Figure S5: NMR spectra of *like*-1-(4-methoxyphenyl)propane-1,2-diol (*like*-**2**).

^{1}H NMR spectrum

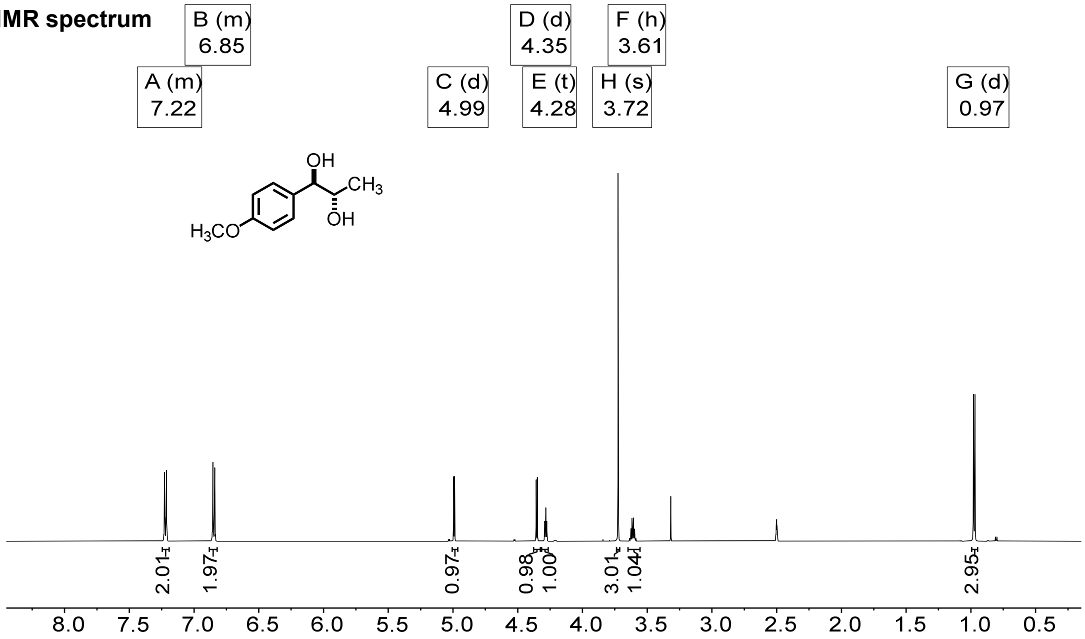

^{13}C NMR spectrum

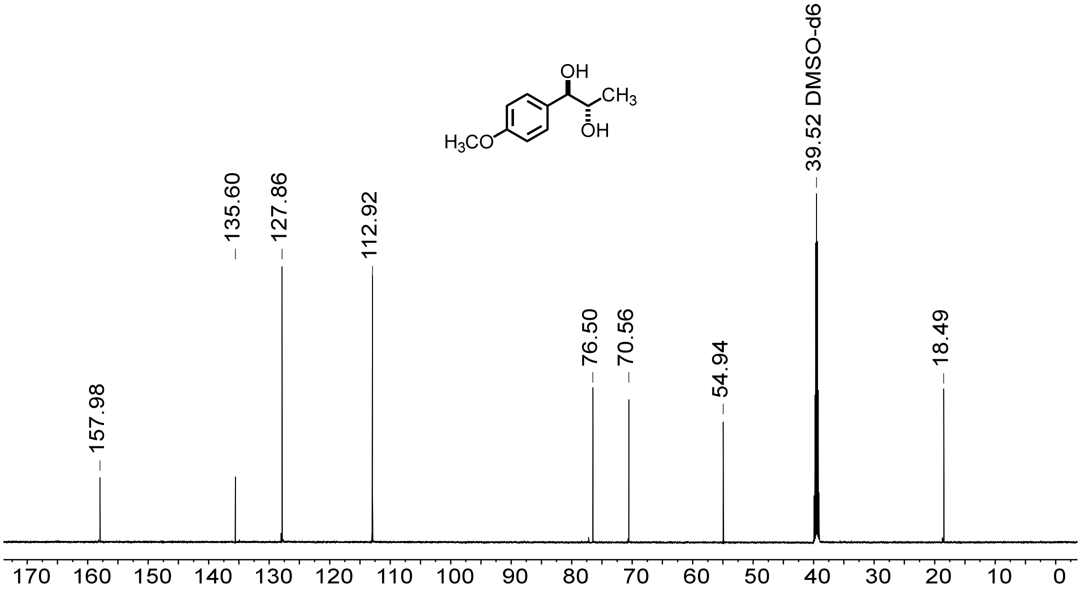

Figure S6: NMR spectra of *unlike*-1-(4-methoxyphenyl)propane-1,2-diol (*unlike*-**2**).

11. References

1. Becke, A. D. Density-functional thermochemistry. III. The role of exact exchange. *J. Chem. Phys.* **1993**, *98*, 5648–5652.
2. Ditchfield, R., Hehre, W. J., Pople, J. A. Self-Consistent Molecular Orbital Methods. IX. Extended Gaussian-type basis for molecular-orbital studies of organic molecules. *J. Chem. Phys.* **1971**, *54*, 724–728.
3. Hehre, W. J., Ditchfield, R., Pople, J. A. Self-Consistent Molecular Orbital Methods. XII. Further extensions of Gaussian-type basis sets for use in molecular-orbital studies of organic-molecules. *J. Chem. Phys.* **1972**, *56*, 2257–2261.
4. Hariharan, P. C. and Pople, J. A. Influence of polarization functions on molecular-orbital hydrogenation energies. *Theor. Chem. Acc.* **1973**, *28*, 213–222.
5. Frisch, M. J., Trucks, G. W., Schlegel, H. B., Scuseria, G. E., Robb, M. A., Cheeseman, J. R., Scalmani, G., Barone, V., Mennucci, B., Petersson, G. A., et al. (2013). Gaussian 09, Revision D.01. Gaussian, Inc., Wallingford CT.
6. Tomasi, J., Mennucci, B., Cammi, R. Quantum Mechanical Continuum Solvation Models. *Chem. Rev.* **2005**, *105*, 2999−3094.
7. Rappe, A. K., Casewit, C. J., Colwell, K. S., Goddard, W. A., Skiff, W. M. UFF, a full periodic table force field for molecular mechanics and molecular dynamics simulations. *J. Am. Chem. Soc.* **1992**, *114*, 10024–10035.
8. Teegardin, K., Day, J. I., Chan, J., Weaver, J. Advances in Photocatalysis: A Microreview of Visible Light Mediated Ruthenium and Iridium Catalyzed Organic Transformations. *Org. Process Res. Dev.* **2016**, *20*, 1156−1163.
9. Montalti, M., Credi, A., Prodi, L., Gandolfi, M. T. (3rd Ed.) (2006). *Handbook of Photochemistry*. CRC Press, Boca Raton. ISBN 978-0824723774.
10. Singh, A., Teegardin, K., Kelly, M., Prasad, K. S., Krishnan, S., Weaver, J. D. Facile synthesis and complete characterization of homoleptic and heteroleptic

cyclometalated Iridium(III) complexes for photocatalysis. *J. Organomet. Chem.* **2015**, *776*, 51–59.

11. Netherton, M. R. and Fu, G. C. Air-Stable Trialkylphosphonium Salts: Simple, Practical, and Versatile Replacements for Air-Sensitive Trialkylphosphines. Applications in Stoichiometric and Catalytic Processes. *Org. Lett.* **2001**, *3* (26), 4295–4298.
